# Metagenomic Insights Into the Diversity of Halophilic Microorganisms Indigenous to the Karak Salt Mine, Pakistan

**DOI:** 10.3389/fmicb.2020.01567

**Published:** 2020-07-14

**Authors:** Leena Mavis Cycil, Shiladitya DasSarma, Wolf Pecher, Ryan McDonald, Maria AbdulSalam, Fariha Hasan

**Affiliations:** ^1^Applied Environmental and Geomicrobiology Laboratory, Department of Microbiology, Quaid-i-Azam University, Islamabad, Pakistan; ^2^Institute of Marine and Environmental Technology, Department of Microbiology and Immunology, University of Maryland School of Medicine, Baltimore, MD, United States; ^3^Yale Gordon College of Arts and Sciences, University of Baltimore, Baltimore, MD, United States; ^4^Institute of Marine and Environmental Technology, Department of Marine Biotechnology, University of Maryland, Baltimore County, Baltimore, MD, United States

**Keywords:** halophiles, halophilic diversity, hypersaline environments, metagenomics, evolution, salt mine, halo-enzymes, halophilic adaptations

## Abstract

Hypersaline regions are terrestrial analogs of the Earth’s primitive ecosystem and extraterrestrial environment. The salt range in Pakistan is considered among a few of the ancient salt deposits in the subcontinent. Karak salt mine is situated at the Northwest end in Pakistan. Despite the fact that halophiles initiated the formation of terrestrial ecosystems, their products and identities remain hidden. Some preliminary studies limited to culture-dependent isolations have been reported. Characterizing the microbiome that spans over centuries of ecosystem development is crucial, given their role in shaping landscape succession and biogeochemical cycles. Here, we used metagenomics techniques to explore the microbial diversity of the Karak salt mine. We used 16S rRNA Illumina amplicon sequencing to characterize the halophilic communities entrapped in Karak mine. The results were interpreted using Illumina Basespace, QIIME, and Cytoscape. Cultures were isolated at 16–25% salinity. Metagenomics data was consistent with our preliminary culturing data, indicating remarkable species to strain-level diversity of unique halophiles. A total of 107,099 (brine) and 122,679 (salt) reads were obtained. 16S rRNA based sequencing revealed a microbiome with bacteria (66% brine and 72% salt) dominated by Bacteroidetes and Proteobacteria with a strikingly high abundance of Archaea (18% brine and 13% salt). Alpha diversity has higher values in salt than in the brine. The study of the halophiles in the Karak salt mine provides clues for species contributing to the maintenance of biogeochemical cycles of the ecosystem. This is the first report of a metagenomic study of any hypersaline region of Pakistan.

## Introduction

It has only recently been accepted that microorganisms commonly inhabit evaporite formations. Microbes in halite crystals serve as excellent models to study evolutionary patterns of ancient microbes because signs such as initial crystallization, surface sterilization, *in situ* observations of the halites and efficient reproducibility of experiments can authenticate the results (DasSarma and DasSarma, 2017). Determining the species colonizing salt crystals, aids in understanding evolutionary patterns and factors influencing microbial survival in paleoenvironments (Jaakkola et al., 2016). Pakistan possesses all three kinds of salt (rock, sea, lake) available on Earth. The hypersaline environments are distributed at the entire length of Pakistan in the form of rock salt, soda lakes, and salt lakes. The salt range in Pakistan represent the most ancient salt deposits on the Asian subcontinent and reveals a series of rock formations ranging from the pre-Cambrian era to the most current geological periods (Cremo, 2001; Jehangiri et al., 2015; Leena et al., 2018). Such exposures are seldom observed and therefore, are remarkable geological phenomena. In the Province of Khyber Pakhtunkhwa (KPK), reserves of salt deposits are found in the mountain range in Karak district at the Northwest end of the Khewra salt range. The salt deposits, also called Kohat rock salt, outcrops the length of 12 km and width of 1/2 km. These mines have unique stratigraphic constraints and fossils, dating its origin millions of years ago. A Pakistan based geological survey assigned the age of Karak salt to the early Eocene period (Roohi et al., 2014; Khattak et al., 2016). This suggests that the microbes entrapped in their salt crystals may have preserved ancient DNA sequences representing the Eocene life. Despite the fact that halophiles may have initiated the formation of terrestrial ecosystems, their products and identities remain enigmatic. A few preliminary studies limited to culture-dependent bacterial isolations and halotolerant enzymes producing bacteria from Karak salt mines have been conducted so far (Roohi et al., 2014; Shah et al., 2017, 2018).

With access to next-generation sequencing, metagenomic approaches have been effectively used to analyze microbial diversity in extreme conditions. They have proven very useful in the reconstruction of the draft and even closed genome sequences of uncultured microorganisms and have recently been used to explore microbial population and composition in diverse hypersaline environments (Mesbah et al., 2007; Ghai et al., 2011; Fernández et al., 2016; Vavourakis et al., 2016). Metagenomic analyses shed light on the interplay between microbial communities and their environment representing ecological diversification. Furthermore, metagenomic studies also allow us to understand the interactions between environmental and evolutionary processes and the role of microbes in the process of nutrient cycling. In this report, we provide the first taxonomic snapshot of microbial communities from the Karak salt mines using metagenomic analysis (Illumina 16S rRNA amplicon sequencing).

### Site Description

**FIGURE 1:**
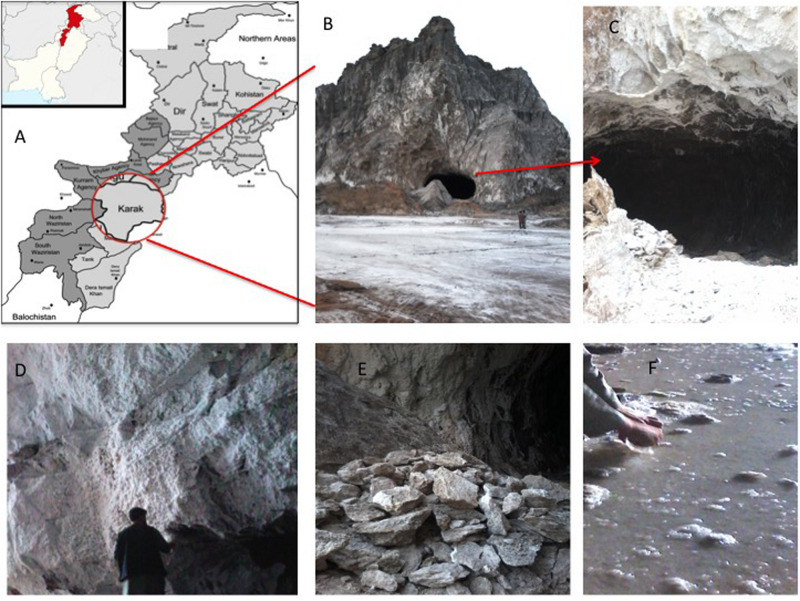
**(A)** Map showing the location of Karak mine in Pakistan; **(B)** entry point of Karak salt mine; **(C)** cave like opening of the Karak salt mine; **(D)** walls of the mine composed of white colored halite; **(E)** grayish-white salt mined from the Karak salt mine; **(F)** saturated Brine pools running within the mine.

The Karak salt mines are located at 32°39′ 0″N, 73°1′ 0″E at an altitude of 678 m/2227 ft. The region consists of rounded dry hills. The rock salts are colored in the shades of light gray to dark gray (Figure 1). Rainfall is meager and intermittent in the area. Also, the environment is dynamic. The region is very hot in summer and very cold in winter (Khan et al., 2011). The geological horizon predicts the age of these rocks as early Eocene (Sharif et al., 2007). The raw salt from mine was recorded to have 0.12% moisture content and 7.98 (mg/Kg) water-insoluble impurities. The composition of salt is fine to medium crystalline, friable and loose, with a slightly sugary texture. The feature distinguishing its halite from the rest of the salt range crystals is its porphyritic nature (Sharif et al., 2007; Nafees et al., 2013; Roohi et al., 2014).

## Materials and Methods

### Sampling and Physiochemical Analysis

Salt and brine samples were collected from the mine under conditions preventing the ingress of exogenous microbial contamination. Brine dripping from the efflorescence of the mine wall and from saturated brine pools was collected in sterilized sampling bottles. The salt crystals were scraped from the mine walls using a sterilized spatula and stored in sterilized bottles and stored in 4°C until further analysis. All samples were sealed and transported to the laboratory (Quaid-i-Azam University, Islamabad) within a few hours there to at 4°C. GPS location coordinates were recorded from each sampling location. The pH and temperature of the brine were determined at the sampling site using pH paper, and later measured under laboratory conditions by a pH meter (Sartorius professional meter PP15). The salinity was measured using a hand-held refractometer and total dissolved solids were also calculated. The chemical analysis of major ions (Sulfates, Chlorides, Phosphates, Bicarbonates) in the brine was performed with Spectroquant (Pharo 100 Spectroquant^®^ Merck, Germany) using standard protocols for the examination of water and wastewater by (APHA) American Public Health Association.

### Reagents and Chemicals

Reagents and chemicals used in this study were of analytical grade and obtained from BDH (Dorset, United Kingdom), Sigma- Aldrich (St. Louis, MA, United States), E Merck (Darmstadt, Germany), Difco Laboratories (Detroit, United States), AVACADO Research Chemicals Ltd (London, United Kingdom).

### Isolation of Halophilic Microorganisms

For our preliminary analysis of detecting culturable halophiles in samples, we used the standard dilution-plating method to count and isolate cultivable microorganisms in salt and brine samples. A series of dilutions (using sterile 1.5 M NaCl solution) was prepared for each sample from the stock suspension, 1 g salt, and l mL brine in 99 mL of 1.5 M NaCl solution. Aliquots, 0.1 mL, from various dilutions (10^–1^ to 10^–4^) were spread on the surfaces of plates containing the solid Nutrient Broth medium (NA) composed of (in grams per liter) (pH 7.0, peptone 5, yeast extract 3, and NaCl (4, 16, and 25%). Solid media were amended with 2% agar. We used the NA medium for the isolation of halophilic bacteria. For haloarchaea, the mineral constituents of the specific medium CM+ have the following composition in (g/L); NaCl: 250, Magnesium Sulfate: 20, (Tri) Sodium Citrate: 3, Bacteriological peptone: 10, Trace metals: 0.1. To solidify the media, 2% agar was added, and the medium pH was adjusted to 7.2. The plates were sealed and incubated at 37°C for 10 days to 2 weeks. Three replicate plates were prepared for each dilution. The total colony numbers were counted, and colony-forming units (CFU) per gram of sample were calculated. Three replicate plates of dilutions containing countable numbers of colonies (100–200) were pooled. Identical colonies (colony shapes, sizes, colors, margins, texture, etc. and cell shapes and sizes as well as the cell Gram stain reaction) were counted, their proportions of the total CFU were calculated, and five replicate representatives were sub-cultured, purified and maintained for further study. For the molecular characterization of the isolates, the genomic DNA was extracted using the ethanol precipitation method (Delbès et al., 2000) with slight modification (Leena et al., 2018). The genomic DNA is confirmed by gel electrophoresis by running it on 1% gel in 0.5 × TBE buffer. Gel images were observed by Bio-Rad gel documentation system. DNA. The 16S rRNA genes in the total genomic DNAs were amplified by polymerase chain reaction (PCR) using the universal primer combination 8F: 5′AGAGTTTGATCCTGGCTCAG and 1492R: 5′AAGTCGTAACAAGGTAACC for bacteria and 4F: 5′TCCG GTTGATCCTGCC and 1492R: 5′AAGTCGTAACAAGGTAA CC for Archaea. Approximately, 5–10 ng of NanoDrop quantified DNA was used for amplifying the entire 16S rRNA with universal primers. The PCR reaction was carried out with PCR mixtures (25 μL) containing 25 pmol of each primer, 1 μL of 100% DMSO, 0.25 μL of 10 mM dNTPs, 0.25 μL of Taq polymerase, 2.5 μL of 10x buffer and 2 μL of the DNA template. The PCR program consisted of an initial denaturation step at 94°C for 5 min; 35 cycles of denaturation at 94°C for 30 s, primer annealing at 55°C for 45 s, and extension at 72°C for 2 min, followed by a final 5 min elongation step at 72°C. Partial sequencing of the 16S rRNA genes was performed using the 3130 XL Genetic Analyzer (BASLab Baltimore, MD, United States). Sequences were subjected to a basic local alignment search tool analysis (BLAST) with the National Center for Biotechnology Information (NCBI) GenBank database. Sequenced 16S rRNA genes data was assembled manually after checking for quality assurance utilizing BioEdit software version 7.3.5. (Hall et al., 2011).

### Culture-Independent Analysis

#### DNA Extraction and Sample Preparation

Environmental DNA was extracted from brine and salt samples. The brine samples were filtered through 0.45 μm mixed cellulose ester (MCE) membranes. The membrane is used for DNA extractions. DNA extraction protocol from salt and filtered brine were carried out by bead beating using the PowerLyzer^®^ PowerSoil^®^ DNA Isolation kit (DNeasy PowerLyzer PowerSoil, QIAGEN Inc., Germantown, MD) with some minor modifications. The alternate lysis protocol that is suggested for hard to lyse cells was used. DNA was quantified using NanoDrop 2000 spectrophotometer (Thermo Fisher Scientific Inc.). Quality was additionally checked by electrophoresis on an Agarose gel (1%), and DNA was stored at -80^o^C. Throughout the DNA extraction, preparation, and sequencing processes, a positive control, and negative control, without DNA were used to monitor for contamination. Approximately, 5–10 ng of NanoDrop quantified DNA was amplified using standard Universal primers provided by Illumina targeting the V3–V4 region of the 16S rRNA gene. The PCR products were then subsequently used for Miseq Illumina sequencing according to the manufacturer’s instructions.

#### Illumina Sequencing

To profile the prokaryotic community, 16S rRNA amplicon sequencing libraries were prepared according to the manufacturer’s instructions (Illumina, San Diego, CA, United States). Briefly, the hypervariable V3–V4 region of the 16S rRNA gene was amplified using the primer pair designed against the surrounding conserved regions (Klindworth et al., 2013). Purified PCR products were pooled in equimolar amounts, and sequenced using the Illumina MiSeq platform (250 bp paired-end reads). The DNA library was sequenced on an Illumina MiSeq platform at the Institute of Marine and Environmental Technology (IMET) using a MiSeq Reagent Kit 300 v2 (Illumina, Baltimore, MD, United States).

#### Sequence Analysis

Raw reads were preprocessed using CLC Genomic Workbench (version 10.1.1) (Qiagen). Adapter sequences were removed and read pairs were quality trimmed (qual. limit = 0.05; ambiguous nucleotide max. = 2; min sequence length = 100 bp) and merged (mismatch cost = 2; gap cost = 3; max. unaligned = 0; min score = 8). Bioinformatics analyses were performed using Quantitative Insights into Microbial Ecology (QIIME), version 1.33, and Illumina Basespace. Open reference OTU picking was performed against the GreenGenes13_8 database (similarity threshold = 0.97; OTU picking method = Uclust; minimum cluster size = 2, however, to generate the OTU network minimum count was kept 10, while OTU picking. OTU networks were generated using the *make_otu_network*.py script. For this analysis, OTUs were re-picked according to the above method, however, the minimum cluster size was increased to 10 in order to reduce the number of nodes. The network was visualized using Cytoscape v3.2.1 using an edge-weighted spring-embedded layout. QIIME pipeline was used to align pair-end reads, assign taxonomy, generating taxonomic plots, and phylogenetic tree. Taxonomies were summarized at multiple levels (L2–L6) using *summarize_taxa.py script*. Representative sequences were aligned with the PyNast algorithm against the Greengenes core set^1^. For comparative analysis, the data is rarefied to an even depth using *single_rarefaction.py* script in QIIME. Using *alpha_diversity.py* script, several diversity and richness metrics were calculated for each community such as observed OTUs including Shannon index for diversity and Chao1 and Simpson richness estimators. The rarefaction plots (Figure 3) were computed at 97% similarity, as part of QIIME’s alpha diversity pipeline. OTU networks were constructed from the output BIOM tables and visualized in Cytoscape (version 3.3.0). Multivariate biome charts for relative abundance were constructed using Krona tools (version: 2.6.1).

## Results

### Analysis of Samples Isolated From Karak Brine

The pH of the brine was 7.14 and the temperature was 28°C at the time of sampling. The salinity of brine measured by a refractometer at 32%, and the Total dissolved solids (TDS) were 35%. Chloride values from brine samples from the Karak mine were estimated as 2002.2 mg/l and sulfates and bicarbonates were measured as 770 and 333 mg/l, respectively.

### Preliminary Data of Isolation and Characterization of Extremely Halophilic Archaea and Bacteria

A total of six halophilic strains were isolated from brine and soil samples, out of which five extreme halophilic bacteria KPS3A, KPS8A, SWM2A, KK1A, and KK2A were selected that showed growth above 16% NaCl concentration and one Archaea was isolated at 25% NaCl, designated KKW1. These selected isolates were then characterized morphologically, and their cellular morphology was observed using phase contrast microscopy. BLASTn results determined the isolates had 16S rRNA gene sequences most similar to *Oceanobacillus* sp., *Oceanobacillus oncorhynchi* subsp. *incaldanensis, Brevibacterium linens, Salicola marasensis, Chromohalobacter salexigens*, and *Haloferax alexandrinus* strains. The sequences were submitted in the NCBI GeneBank with the following accession numbers *KPS3A*-MT406254, *KPS8A*-MT406253, *SWM2A*-MT406257, *KK1A-*MT406258, *KK2A*-MT406255, and *KKW1*-MT406256, respectively, where each designated specie is followed by its respective accession number (Table 1).

**TABLE 1:** Morphological and molecular characterization of isolates.

S. No.	Isolate	Media (%NaCl) at 37°C	Cellular morphology	Incubation time (days)	Colony size (mm)	Colony morphology	16S rRNA characterization	NCBI accession numbers
1	KPS3A	16 NA	Spore forming rods	3	3–4	Off white, watery and sticky, round	*Oceanobacillus* sp.	*MT406254*
2	KPS8A	16 NA	Rods, spores, motile	4	1–2	White, opaque, and round	*Oceanobacillus oncorhynchi* subsp. *incaldanensis*	*MT406253*
3	SWM2A	16 NA	Short rods, no spores, no motility	4	1–2	Yellow, opaque, and round	*Brevibacterium linens/aureum*	*MT406257*
4	KK1A	16 NA	Short rods, motile	7	1	Light yellow, round, transparent, and watery	*Salicola marasensis*	*MT406258*
5	KK2A	16 NA	Cocco-baciili	7	1	Whitish, transparent, moist and round	*Chromohalobacter salexigens*	*MT406255*
6	KKW1	25 CM+	Pleomorphic	14	3–4	Pale pink, slimy, and moist	*Haloferax alexandrines*	*MT406256*

### Culture-Independent Studies

16S rRNA reads generated from both brine and salt datasets were used to build a representation of the high-level phylogenetic groups in each dataset using QIIME. Both datasets display a remarkably diverse collection of taxonomic clusters. The outcomes of all methods of classification were largely in agreement and showed exciting merits.

#### Statistics

**FIGURE 2:**
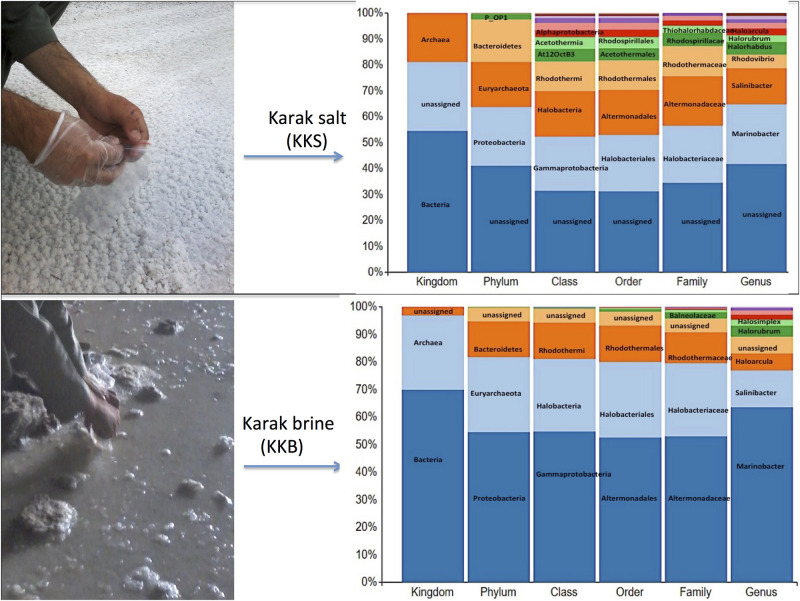
The multi-level column-chart demonstrating the relative abundance of the top 10 classification within each taxonomic level in Karak brine and salt samples. The unassigned reads at each level is presented as “other.”

**FIGURE 3:**
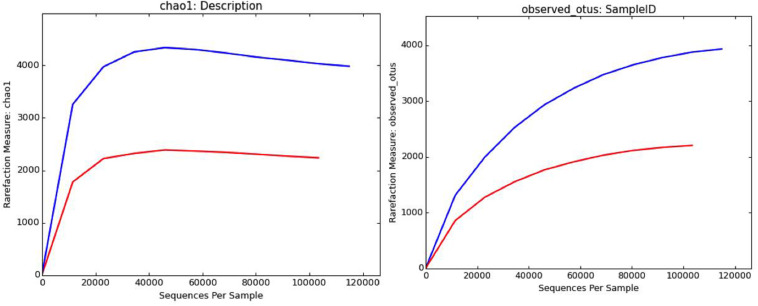
Rarefaction curves computed through QIIME depicting distribution and diversity of microbial communities in salt (blue) and brine (red) samples using Chao1 richness estimator and observed OTUs at 97% sequence similarity.

The brine (KKB) and salt (KKS) samples from the Karak salt mines were subjected to high-throughput sequencing analyses. Sequences acquired after the quality filtering were trimmed, and only sequences of high quality were preserved for further analysis. A total of 229,778 high-quality prokaryotic reads were obtained. After the rarefaction of data to an even depth, the analysis presented a total of 5491 operational taxonomic units (OTUs) using ≥97% sequence identity. Interestingly, salt sample KKS (3911 OTUs), corresponding to brine sample KKB (2234) contained a higher number of distinct OTUs, and a total of 635 shared OTUs were detected between both samples. KKS-salt contains the highest number of unclassified OTUs (27%). We recovered 46 classes, 77 orders, 106 families, and 156 genera across both sampling sites, from reads categorized at each taxonomic level. For both sample datasets, over 80% of all quality reads were constantly categorized at the Phylum level. However, about 16.3% of reads remained unclassified (Figure 2). Alpha diversity was measured using metrics such a Good’s coverage, Shannon index, Simpson, and nonparametric Chao1 estimator (Table 2). The results indicated that between the two samples, KKS-salt had the highest diversity as compared to KKS-brine as demonstrated by Shannon indices of 7.11 and 5.55, respectively. Due to the elimination of singletons, the number of observed OTUs was observed to be very close to the measured richness values using the indices mentioned above. The results of Good’s coverage (99% coverage), and rarefaction curves (Figure 3) suggest an almost complete sampling. A comparison of the rarefaction curves with the number of OTUs determined by the Chao1 richness estimator and other diversity indices revealed that the samples have significant microbial diversity.

**TABLE 2:** Alpha diversity and richness estimators calculated for Operational Taxonomic Units (OTUs) of environmental samples, clustered at 97% similarity.

Diversity metrics	KKB (Karak brine)	KKS (Karak salt)
Chao1	2234.46	4054.72
Simpson	0.90	0.97
Observed_otus	2215	3911
Goods_coverage	0.99	0.99
Shannon	5.55	7.11

#### Community Structure

**FIGURE 4:**
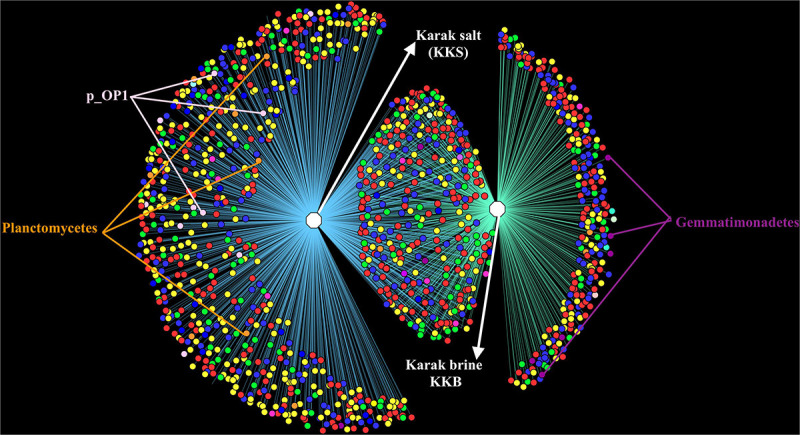
OTU Network demonstrating the number of unique and shared OTUs in the brine and salt samples of Karak mine. The OTU table was generated using 97% cut-off sequence value and mc-10. The taxonomic clustering was displayed in the form of OTU network using the edge-weighted, spring embedded layout in Cytoscape. Node color denotes consensus taxonomy (phylum). Edges connecting OTU and sample indicate the presence of the OTU in that sample.

**FIGURE 5:**
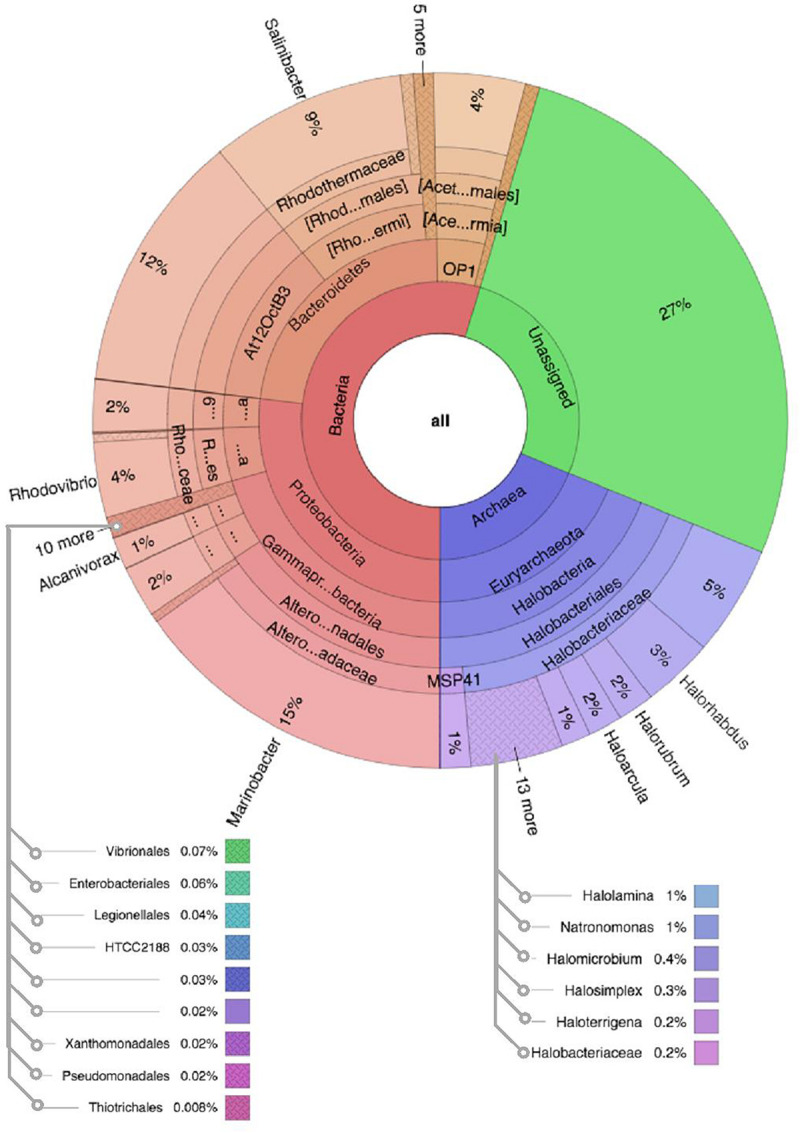
Multivariate OTU clusters of Karak salt samples. Legends: Archaea (blue), Unassigned (green), and Bacteria (Red/Orange), with lighter shades signifying lower taxonomic levels (toward species-level).

**FIGURE 6:**
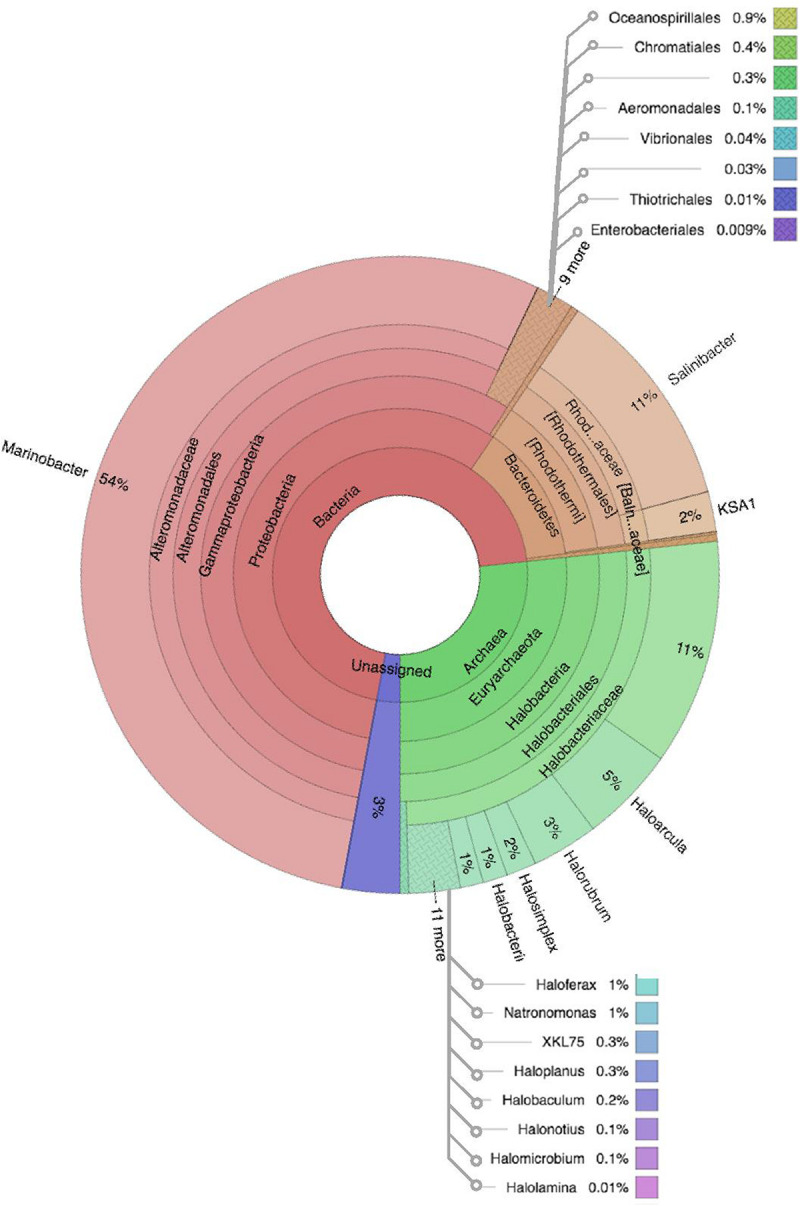
Multivariate OTU clusters of Karak brine samples. Legends: Archaea (blue), Unassigned (green), and Bacteria (Red/Orange), with lighter shades signifying lower taxonomic levels (toward species-level).

Comparative analysis of both the brine (KKB) and salt (KKS) datasets at the Phylum level showed that 27% reads from the KKS-salt sample and 3% of the reads are from KKB-brine sample remain could not be assigned to any Phylum. For both the datasets, the microbial community comprised of 60% of bacteria and 22% of archaea. A total of 28 bacterial phyla were identified in both the Karak salt and brine samples. The top ten abundant phyla contributed up to 90% of the total diversity. Remarkably, Proteobacteria (40.6%) remain as the dominant group followed by Euryarchaeota (22.8%) and Bacteroidetes (17.8%), Candidate division OP1 (2.10%), Planctomycetes, Firmicutes, and Gemmatimonadetes. Actinobacteria, Cyanobacteria, and Chloroflexi represented a comparatively minor percentage of total diversity. OTU network depicts the large number of unassigned OTUs in the salt samples (Figure 4). Euryarchaeota contributed 100% to the total archaeal diversity. At the genus level, 73 and 95% of the 16S rRNAs reads were assigned to KKB-brine and KKS-salt datasets, respectively. The archaea were dominated by numerous clades of Halobacteria that dwell in salt lakes and salterns and can propagate on or within salt crystals. Many archaeal OTUs between the two samples are shared. However, a significant number of unique archaea were detected in the sample with high salinity (KKS-salt) (Figure 4). The most abundant members of Halobacteria in our dataset were the species *Haloarcula* (3% reads), followed by *Halorubrum* (2.5%) and *Halorhabdus* (2%). *Halorhabdus* was detected in considerable abundance across KKS (3.1) compared to KKB (0.8%). Archaeal OTUs representing nine genera within the Class *Halobacteria (Halobacterium, Haloarcula, Halorubrum Halonotius, Halorhabdus, Haloplanus, Natronomonas Halobacteriaceae*, and *Halolamina)* were shared across samples. *Halobaculum* and *Haloterrigena* were exclusive to Karak salt whereas *Haloferax* and *Halosimplex* were only observed in Karak brine (Supplementary Figure S1). Phylum Proteobacteria were ubiquitous across both samples. A comparatively higher abundance of this Phylum was observed across the KKB-brine sample (54.7%) as compared to the KKS-salt (26.5%). Among the Proteobacteria, Alpha-, Beta-, Gamma-, and Delta-Proteobacteria were detected in all samples, out of which 91% of members belong to Gamma-proteobacteria and represents the most abundant members (20.2–54.3%) across both salt and brine samples, respectively. The second most abundant Phyla Bacteroidetes consists of two classes Rhodothermi and At12OctB3 forming 11.28 and 5.99% of the total community. Phylum Bacterioidetes was detected in higher abundance in salt sample KKS (22.4%) as compared to brine sample KKB (13.1%). Similarly, members of Phyla Planctomycetes and Firmicutes were detected in salt sample KKS. Members of Candidate division OP1 were present exclusively in salt samples (4.98%). The most abundant bacterial OTUs detected in the datasets were *Marinobacter* (41.4%), *Salinibacter* (10.9%) (Figures 5, 6).

## Discussion

To fully comprehend the role that microorganisms play in Earth’s Subsurface environment, it is essential to study the life entrapped in the closed environment of the salt mine. Microbes in salt crystals have implications for the preservation of life in the fluid trapped in the salt crystals at hypersalinity (Adamski et al., 2006; Pasteris et al., 2006). Salt crystals from multiple environments such as salt mines; salt plains, rock salts, and food-grade salts have been shown to harbor viable, ancient microbes (Vreeland et al., 1998; Caton et al., 2009; Henriet et al., 2014; Robinson et al., 2015). However, to the best of our knowledge, metagenomic community profiles of halophiles from these salt mines have not been reported. Hence, in this study, we present for the first time the novel insights into the community structure of indigenous microbiota of Karak salt mines by using metagenomics, which provides a snapshot of microbial community diversity, distribution, and functionality.

The salt crystals of Karak salt mine are reported as 98% pure (halite). The salinity of the brine pools running within the mine runs up to 32%, hence reaching close to the higher range of salinity of brines from Andean salares which had also been investigated for prokaryotic diversity (Maturrano et al., 2006; Haferburg et al., 2017). In this study, isolations were performed at near-neutral pH and temperature ranging from 37°C in accordance with the conditions measured at the investigated site. Our results on cultivable halophiles from Karak salt mine coincide with a few studies conducted over the years to isolate halophiles from the rock salts starting from isolations from the Permian salt mine, Kansas (Reiser and Tasch, 1960). Recent isolations of halophiles were reported from Great Salt Plains of Oklahoma (Caton et al., 2009) Death Valley California (Mormile et al., 2003; Ram et al., 2016) China, Bolivia (DasSarma et al., 2019) and Anatolia, Turkey (Yildiz et al., 2012; Xiao et al., 2013). In Pakistan, only a few studies regarding bacterial isolations from saline environments have been conducted, reporting the dominance of genus Bacillus in the rock salt along with *Planococcus, Halomonas, Brevibacterium, Kocuria, Salinivibrio, Salinicoccus*, and *Kushneria* (Muyzer et al., 1993; Akhtar et al., 2008; Roohi et al., 2014). Our preliminary isolation study was focused on the isolation of extreme halophiles (both archaea and bacteria) so that in addition to earlier reports of isolation, which comprise of mostly, slight to moderate halophiles, our report contributes in capturing the extreme halophilic culturable microbial community of the rock salt deposits of Karak mine. Isolates *Chromohalobacter (KK2) Haloferax (KKW1)* and *Oceanobacillus* sp. (*KPS3A* and *KPS8A*) were also detected in the metagenomic data. In this investigation, isolation yielded several species such as *Haloferax* and *Salicola* that probably belong to the unique biodiversity that is not adequately present to be identified by metagenomics but may be opportunists and effortlessly grow under the applied cultivation conditions. Many genera that are detected by metagenomics, may not be isolated soon due to specie’s yet unknown growth requirements (Bolhuis et al., 2004; Naghoni et al., 2017). To attain a better comparative evaluation between cultivation approaches and metagenomics, a multitude of isolates would be required to grow under variable conditions.

The metagenomic technique serves as a powerful tool to study microbial diversity directly from the environment and also allows the exploration of novel microorganisms and their adaptation mechanisms in relatively unexplored extreme habitats (Behzad et al., 2016). Miseq-Illumina analysis showed the dominance of Proteobacteria and Bacteroidetes with a strikingly high abundance of Archaea. Members of these taxa were formerly verified in other hypersaline and tropical soda lake ecosystems such as Salar de Uyuni, Lake Nakuru, Lake Bogoria, Kae Magadi as well as from the Lonar Lake ecosystem (Duckworth et al., 1996; Margesin and Schinner, 2001; Vargas et al., 2004; Kambura et al., 2016; Paul et al., 2016). The Karak salt mine has a metabolically diverse halotolerant bacterial population. Members of Proteobacteria phylum were found to be the principal inhabitants of the Karak mine, covering almost half of the bacterial OTUs (48.3%) within the data. Within Phylum Proteobacteria, members of the Altermonadaceae, Rhodospirillaceae, Ectothiorhodospira, Oceanospirillaceae, and Thiohalorhabdaceae were found to be prevalent in our taxonomic review and had also been independently verified in such ecosystems using culture-based methods (Chandrasekhar et al., 2002; Paul et al., 2016). These bacteria are known for their metabolic diversity and ubiquitous nature, which allows them to survive in extreme environments by utilizing various carbon compounds to preserve both aerobic and anaerobic lifestyles (Janssen, 2006). OTUs belonging to the Phylum Bacteroidetes amount up to ∼18% of the total reads. The decreased number of reads associated with anoxygenic photosynthetic (sulfur and non-sulfur purple, and green bacteria) in Karak samples at high salinity was consistent with other reports. However, further study is needed to determine the impact of these groups of Archaea to the primary productivity in the salt mines samples. Investigation of the average community composition of the brine and salt samples showed that the most greatly represented genus was *Marinobacter* which contributed up to 23.6% of the total data followed by *Salinibacter* (10%), *Halorubrum* (3%), *Haloarcula* (2.5%), and *Halorhabdus* (2.1%). Within Archaea, clear predominance of the Class, Halobacteria was observed in Karak mine samples. A massive number of the archaeal OTUs (22.8%) were detected in the data. Among these, 12% showed high-sequence similarity with the identified archaeal genera (Burns et al., 2004; Dillon et al., 2009; Ventosa et al., 2014; Ruvindy et al., 2016). However, the number of novel archaeal genera (8.3%) was noteworthy.

All of the archaeal sequences fell into the Class Halobacteria. In the present study, *Halorubrum* was found to be the most abundant genus in brine, and *Haloarcula* was the most abundant genus in salt samples suggesting their significant roles in their respective habitat. Abundant strains of the genera *Halorubrum* and *Haloarcula* in the halite crystals have also been observed in other culture-based studies of salt mines in China and food-grade salt crystals (Gibtan et al., 2017). *Halorhabdus* is the most extreme halophilic member of the family Haloarculaceae and is present in much more abundance in salt (3.3%) than in brine (0.8%). Other archaeal genera such as *Halobaculum* (0.6%) and *Halotinus* (0.6%) were also found in relatively more abundance in the salt sample. Members of genus *Halonotius* are reported to be ubiquitously present in highly saline environments (Durán-Viseras et al., 2019). However, *Haloquadratum*, which is one of the most highly represented archaeal genera in salterns (Welsh, 2000; Oren, 2012; Dillon et al., 2013) appeared to be absent in the Karak mine samples. *Haloquadratum* was also reported as an insignificant member in commercial salts suggesting its role limited to hypersaline pond systems and salterns. It has previously been suggested that widely used 16S rRNA-targeted primer sets may not be suitable to indicate the presence of Nanohaloarchea and so using archaeal specific primers could probably provide a more in-depth diversity analysis (Narasingarao et al., 2012). The diversity of microbes within the sediment and water samples was distinct concerning community composition. The OTU network generated from the16S rRNA-based metagenomic analysis of the assembled reads verified the overlap in the diversity between the brine and salt samples. The overlap in sequences mostly consists of Archaea, Bacteriodetes, and Proteobacteria. Candidate Div. OP1, Firmicutes, and Planctomycetes were exclusively noted in the salt samples, while Gemmatimonadetes were found chiefly in the brine samples. The diverse patterns of microbial community assemblage across salt and water are likely to be caused by variances in salinity, nutrient availability, and redox state in this ecosystem.

The most highly represented bacterial OTUs belonged to genera *Marinobacter*. It was found to be the most abundant genus (35%) in both salt crystals and brine samples. Its presence in rock salts has also been described in the salt mines of China, Romania, and Turkey, as well as in the food-grade salts (Carpa et al., 2014; Henriet et al., 2014; Cınar and Mutlu, 2016; Haferburg et al., 2017). Members belonging to *Marinobacter* family were found in diverse environments such as saline soils, petroleum field brines, surface seawater, ballast water (Arctic), marine snow, hydrothermal plumes, hot springs at coasts, deep seawater, volcanic basalts and beneath the iron ranges of Minnesota (Singer et al., 2011; Handley and Lloyd, 2013; Bonis and Gralnick, 2015). Subsequently, the strains show diverse potential to survive poly extreme conditions such as heavy metal, antibiotic resistance, phage resistance, Fe (II) oxidation, and electrogenic Perchlorate-Reduction allowing for active exploitation of an unstable environment (Stepanov et al., 2016). *Marinobacter* strains have substantial metabolic versatility, which makes them attractive for biotechnological exploitation. The dominance of *Marinobacter* in the Karak salt mines sheds light on its significant contribution to the maintenance of the ecosystem. Hypersaline environments are considered as the terrestrial analogs of the extra-terrestrial environment. Given the discovery of perchlorate among the salts detected by the Phoenix Lander on Mars, an abundance of *Marinobacter* in Karak salt mines, its perchlorate reducing ability and its ability to survive under poly-extreme conditions implies that presence of perchlorate does not preclude the prospect of halophilic life on Mars. This argument is also supported by the abundance of other perchlorate-resistant halophiles in the salt samples such as [*Haloarcula, Haloferax*, *Halobacterium*, *Halorubrum* (Laye and DasSarma, 2018) and the bacterium *Halomonas elongata*] (Oren, 2014). Hypersaline habitats have been known as a sanctuary to preserve biological material on time periods exceeding that of non-saline systems. Thus, the presence of paleo-sulfate lakes on Mars is of particular significance for astrobiology, as these might have preserved signs of previous microbial inhabitation on Mars (Carrier et al., 2020). Further biological characterization of the Karak mines is essential to define the limits to microbial growth in these hypersaline environments. With a relative abundance of 10%, the genus *Salinibacter* represents the second most abundant extremely halophilic member thriving in the brine sample. The genus is distributed globally in hypersaline environments (Oren, 2013).

### Taxa With Known Ecological Roles

In addition to salt tolerance, a number of geochemically relevant species were detected in our data. A low abundance of anoxygenic phototrophs (Chloroflexi, Chlorobi, Rhodobacterales, and Chromatiales) was detected. The low abundance of phototrophs implies that in the Karak mine, particularly in salt samples, carbon is fixed using alternative pathways. Perhaps little known and relatively uncharacterized phylogenetic groups, exclusive to salt crystals such as the Planctomycetes, Firmicutes, Acetothermia, and Euryarchaeota, are fulfilling this significant role. Acetothermia belonging to Candidate div OP1 are currently uncultivated but has been identified in a variety of environments using culture-independent methods. One report revealed genes encoding for the (acetyl-CoA) pathway for CO_2_ fixation in Candidatus “Acetothermum autotrophicum.” The presence of genes for CO_2_ fixation in Acetothermia suggests that they could contribute to primary production through chemolithoautotrophic acetogenesis (Farias et al., 2017). The sulfur cycle seemed more active in the salt samples as compared to brine with the presence of *Thiohaldorhabdus, Halorhabdus*, and *Halorhodospira* involved in sulfur cycling. Members of family Desulfohalobiaceae (Deltaproteobacteria) dealing with the reductive part of the sulfur cycle were present in less abundance. These are reported to be more active at lower salinity (Lane, 1991; Jackson et al., 2014). Among Gammaproteobacteria, *Thiohalorhabdus* and members from order Chromatiales such as *Halorhodospira* grow by autotrophically utilizing energy derived from the oxidation of elemental sulfur and reduced inorganic sulfur compounds. The presence of both sulfur-reducing and sulfur-oxidizing prokaryotes indicates the presence of an active sulfur cycle, especially in the salt samples. The presence of the nitrogen cycle was also detected with the presence of OTUs affiliated with efficient ammonia-oxidizing bacteria (Beta-proteobacteria) *Nitrosomonas*. These chemolithotrophic ammonia-oxidizing bacteria AOB commonly belong to the Beta- and Gammaproteobacteria (Purkhold et al., 2000). Phosphate solubilizing archaea, *Haloarcula, Halobacterium*, and *Halolamina* were observed in our dataset. These organisms had implicated involvement in the maintenance of Phosphorous cycle in Hypersaline environments (Yadav et al., 2015). *Marinobacte*r representing the major genera in Karak samples was also reported to have incredible metabolic potential and had shown capabilities of anaerobic respiration using dimethylsulfoxide (DMSO) or nitrate (Yau et al., 2013).

### Diversity Indices

Alpha diversity was measured using metrics such a Good’s coverage, Shannon index and specie richness were calculated with a nonparametric Chao1 estimator (Table 2). With a number of 3956 OTUs the salt samples of Karak mine (KKS) displayed the more diverse habitat whereas the brine KKB with 2215 OTUs showed a somewhat lower diversity. The diversity metrics analysis indicated that between the two samples, KKS (salt) had the highest diversity as compared to KKS (brine). The results of Good’s coverage (99% coverage), and rarefaction curves (Figure 3) suggest an almost complete sampling. Perhaps, the higher abundance of different minerals in halite salt as compared to water samples acts as an abiotic regulator on microbial community composition (Paul et al., 2016). The statistics revealed a high proportion of co-shared phylotypes (635) between the salt and brine samples, signifying the presence of a well-adapted and stable halophilic core community distribution. However, unique OTUs in salt samples such as Acetothmeria and Planctomycetes highlights their specific role in the survival and functioning of microbial community entrapped within salt crystals.

## Conclusion

The current examination provides first-ever insight into the composition of halophilic microbial communities from an uncharacterized ecosystem of Karak salt mine, Pakistan. The significant existence of halophiles advocates the capacity of microbes to grow and divide in this extreme environment. The distribution of archaeal and bacterial communities in the Karak salt mines is diverse, with the members of Phyla Euryarchaeota, Bacteroidetes, Proteobacteria, Firmicutes, and Class Acetothermia as top 5 phyla. Representatives of the genera *Marinobacter* and *Salinibacter* were the principal inhabitants in samples suggesting their crucial role in this environment. In addition to the adaptation to hypersalinity, the perchlorate-resistant ability of bacteria (*Marinobacter, Halomonas*) and archaea (*Haloarcula, Haloferax*) dominating the salt mine, make this environment and isolates the best model for astrobiological studies. Although the uncharacterized OTUs covered a relatively large proportion of the overall population in our analysis, the number of novel genera spotted revealed significant diversity, suggesting that a more detailed examination needs to be performed to characterize them. Future research with multiple sampling points, e.g., halite crystals from multiple-layered sediments and at different time periods within the mine will increase our understanding of the ecology of extreme halophiles. Our future prospects include a detailed analysis of culturable diversity for a more comprehensive snapshot of microbial diversity. The metagenome analysis cannot calculate protein synthesis and gene expression. Therefore, in order to investigate the functional complexity in microbial ecosystems, a metatranscriptomic method is essential. Analogous halites on Mars would signify an excellent setting to investigate for conserved organic material and associated biomarkers, which might be concentrated through evaporative processes and subsequently conserve life that could persist over geologic time.

## Data Availability Statement

The datasets generated for this study can be found in the https://www.ncbi.nlm.nih.gov/Traces/study/?acc=PRJNA483802. Additional data for 16S rRNA sequences of isolates in this study can be found at: https://www.ncbi.nlm.nih.gov/nuccore/?term=MT406253:MT406258[accn].

## Author Contributions

LC drafted the manuscript, performed all laboratory experiments, did downstream analysis with the results, and performed bioinformatics analysis of the raw RNA-seq data using the transcriptomic pipeline. FH and SD provided experimental guidance and manuscript feedback and editing. RM, MA, and WP provided guidance and critical feedback and editing of the manuscript. All authors contributed to the article and approved the submitted version.

## Conflict of Interest

The authors declare that the research was conducted in the absence of any commercial or financial relationships that could be construed as a potential conflict of interest.

## Footnotes

1 http://greengenes.lbl.gov

## Supplementary Material

FIGURE S1 Relative distribution of halophilic communities in brine and salt samples of Karak salt mine plotted at low-level tax. Sequences that were not allocated to any phylum are depicted as “unassigned.”

## References

[B1] Adamski J. C. Roberts J. A. Goldstein R. H. (2006). Entrapment of bacteria in fluid inclusions in laboratory-grown halite. *Astrobiology* 6 552–562. 10.1089/ast.2006.6.552 16916282

[B2] Akhtar N. Ghauri M. A. Iqbal A. Anwar M. A. Akhtar K. (2008). Biodiversity and phylogenetic analysis of culturable bacteria indigenous to Khewra salt mine of Pakistan and their industrial importance. *Braz. J. Microbiol.* 39 143–150. 10.1590/S1517-838220080001000029 24031194 PMC3768371

[B3] Behzad H. Ibarra M. A. Mineta K. Gojobori T. M. (2016). Metagenomic studies of the red sea. *Gene* 576 717–723. 10.1016/j.gene.2015.10.034 26526132

[B4] Bolhuis H. Poele E. M. Rodrıguez-Valera F. (2004). Isolation and cultivation of Walsby’s square archaeon. *Environ. Microbiol.* 6 1287–1291. 10.1111/j.1462-2920.2004.00692.x 15560825

[B5] Bonis B. M. Gralnick J. A. (2015). *Marinobacter subterrani*, a genetically tractable neutrophilic Fe (II)-oxidizing strain isolated from the Soudan Iron Mine. *Front. Microbiol.* 6:719. 10.3389/fmicb.2015.00719 26236300 PMC4503921

[B6] Burns D. G. Camakaris H. M. Janssen P. H. Dyall-Smith M. L. (2004). Combined use of cultivation-dependent and cultivation-independent methods indicates that members of most haloarchaeal groups in an Australian crystallizer pond are cultivable. *Appl. Environ. Microbiol.* 70 5258–5265. 10.1128/AEM.70.9.5258-5265.200 15345408 PMC520848

[B7] Carpa R. Keul A. Muntean V. Dobrotã C. (2014). Characterization of halophilic bacterial communities in Turda salt mine (Romania). *Orig. Life Evol. Biosph.* 44 223–230. 10.1007/s11084-014-9375-4 25476992 PMC4669543

[B8] Carrier B. L. Beaty D. W. Meyer M. A. Blank J. G. Chou L. DasSarma S. (2020). Mars extant life: what’s next? conference report. *Astrobiology* 20 785–814. 10.1089/ast.2020.2237 32466662 PMC7307687

[B9] Caton T. M. Caton I. R. Witte L. R. Schneegurt M. A. (2009). Archaeal diversity at the Great salt plains of Oklahoma described by cultivation and molecular analyses. *Microb. Ecol.* 58 519–528. 10.1007/s00248-009-9507-y 19306116 PMC4066810

[B10] Chandrasekhar D. V. Mishra D. C. Rao G. P. Rao J. M. (2002). Gravity and magnetic signatures of volcanic plugs related to Deccan volcanism in Saurashtra, India and their physical and geochemical properties. *Earth Planet. Sci. Lett.* 201 277–292. 10.1016/s0012-821x(02)00712-4

[B11] Cınar S. Mutlu M. B. (2016). Comparative analysis of prokaryotic diversity in solar salterns in eastern Anatolia (Turkey). *Extremophiles* 20 589–601. 10.1007/s00792-016-0845-7 27306996

[B12] Cremo M. A. (2001). “Paleobotanical anomalies bearing on the age of the salt range formation of Pakistan, a historical survey of an unresolved scientific controversy,” in *Proceedings of the XXIst International Congress of History of Science*, Mexico.

[B13] DasSarma P. Anton B. P. DasSarma S. L. Martinez F. L. Guzman D. Roberts R. J. (2019). Genome sequences and methylation patterns of natrinema versiforme BOL5-4 and natrinema pallidum BOL6-1, Two extremely halophilic archaea from a bolivian salt mine. *Microbiol. Resour. Announc.* 8:e00810-19. 10.1128/MRA.00810-19 31416876 PMC6696651

[B14] DasSarma S. DasSarma P. (eds) (2017). “Halophiles,” in *eLS*, Vol. 1(Chichester: Wiley), 1–13.

[B15] Delbès C. Moletta R. Godon J. J. (2000). Monitoring of activity dynamics of an anaerobic digester bacterial community using 16S rRNA polymerase chain reaction–single-strand conformation polymorphism analysis. *Environ. Microbiol.* 2 506–515. 10.1046/j.1462-2920.2000.00132.x 11233159

[B16] Dillon J. G. Carlin M. Gutierrez A. Nguyen V. McLain N. (2013). Patterns of microbial diversity along a salinity gradient in the Guerrero Negro solar Saltern, Baja CA Sur, Mexico. *Front. Microbiol.* 4:399. 10.3389/fmicb.2013.00399 24391633 PMC3868825

[B17] Dillon J. G. Miller S. Bebout B. Hullar M. Pinel N. Stahl D. A. (2009). Spatial and temporal variability in a stratified hypersaline microbial mat community. *FEMS Microbiol. Ecol.* 68 46–58. 10.1111/j.1574-6941.2009.00647.x 19175677

[B18] Duckworth A. W. Grant W. D. Jones B. E. Van Steenbergen R. (1996). Phylogenetic diversity of soda lake alkaliphiles. *FEMS Microbiol. Ecol.* 19 181–191. 10.1111/j.1574-6941.1996.tb00211.x

[B19] Durán-Viseras A. Andrei S. Ghai R. Sánchez-Porro C. Ventosa A. (2019). New *Halonotius* species provide genomics-based insights into cobalamin synthesis in haloarchaea. *Front. Microbiol.* 10:1928. 10.3389/fmicb.2019.01928 31507553 PMC6719526

[B20] Farias M. E. Rasuk M. C. Gallagher K. L. Contreras M. Kurth D. Fernandez A. B. (2017). Prokaryotic diversity and biogeochemical characteristics of benthic microbial ecosystems at La Brava, a hypersaline lake at Salar de Atacama, Chile. *PLoS One* 12:e0186867. 10.1371/journal.pone.0186867 29140980 PMC5687714

[B21] Fernández A. B. Rasuk M. C. Visscher P. T. Contreras M. Novoa F. Poire D. G. (2016). Microbial diversity in sediment ecosystems (Evaporites Domes, microbial mats, and crusts) of Hypersaline Laguna Tebenquiche, Salar de Atacama, Chile. *Front. Microbiol.* 7:1284. 10.3389/fmicb.2016.01284 27597845 PMC4992683

[B22] Ghai R. Pasic L. Fernández A. B. Martin-Cuadrado A. B. Mizuno C. M. McMahon K. D. (2011). New abundant microbial groups in aquatic hypersaline environments. *Sci. Rep.* 1:135. 10.1038/srep00135 22355652 PMC3216616

[B23] Gibtan A. Park K. Woo M. Shin J. Lee D. Sohn J. H. (2017). Diversity of extremely halophilic archaeal and bacterial communities from commercial salts. *Front. Microbiol.* 8:799. 10.3389/fmicb.2017.00799 28539917 PMC5423978

[B24] Haferburg G. Gröning J. A. D. Schmidt N. Kummer N. Erquicia J. C. Schlömann M. (2017). Microbial diversity of the hypersaline and lithium-rich Salar de Uyuni, Bolivia. *Microbiol. Res.* 199 19–28. 10.1016/j.micres.2017.02.007 28454706

[B25] Hall T. Biosciences I. Carlsbad C. (2011). BioEdit: an important software for molecular biology. *GERF Bull. Biosci.* 2 60–61.

[B26] Handley K. M. Lloyd J. R. (2013). Biogeochemical implications of the ubiquitous colonization of marine habitats and redox gradients by *Marinobacter* species. *Front. Microbiol.* 4:136. 10.3389/fmicb.2013.00136 23734151 PMC3660661

[B27] Henriet O. Fourmentin J. Delincé B. Mahillon J. (2014). Exploring the diversity of extremely halophilic archaea in food-grade salts. *Int. J. Food Microbiol.* 191 36–44. 10.1016/j.ijfoodmicro.2014.08.019 25217724

[B28] Jaakkola S. T. Ravantti J. J. Oksanen H. M. Bamford D. H. (2016). Buried alive: microbes from ancient halite. *Trends Microbiol.* 24 148–160. 10.1016/j.tim.2015.12.002 26796472

[B29] Jackson K. L. Whitcraft C. R. Dillon J. G. (2014). Diversity of desulfobacteriaceae and overall activity of sulfate-reducing microorganisms in and around a salt pan in a Southern California coastal wetland. *Wetlands* 34 969–977. 10.1007/s13157-014-0560-z

[B30] Janssen P. H. (2006). Identifying the dominant soil bacterial taxa in libraries of 16S rRNA 16S rRNA genes. *Appl. Environ. Microbiol.* 72 1719–1728. 10.1128/aem.72.3.1719-1728.2006 16517615 PMC1393246

[B31] Jehangiri M. Hanif M. Arif M. Jan I. F. Ahmad S. (2015). ‘The early Cambrian Khewra sandstone, salt range, Pakistan, endorsing southern Indian provenance’. *Arab. J. Geosci.* 8 6169–6187. 10.1007/s12517-014-1649-7

[B32] Kambura A. K. Mwirichia R. K. Kasili R. W. Karanja E. N. Makonde H. M. Boga H. I. (2016). Bacteria and Archaea diversity within the hot springs of Lake Magadi and Little Magadi in Kenya. *BMC Microbiol.* 16:136. 10.1186/s12866-016-0748-x 27388368 PMC4936230

[B33] Khan M. Musharaf S. Shinwari Z. K. (2011). Ethnobotanical importance of halophytes of Noshpho salt mine, District Karak, Pakistan. *Res. Pharm. Biotechnol.* 3 46–52.

[B34] Khattak N. U. Khan A. M. Ali N. Ahmed F. Tahir Shah M. (2016). Recognition and characterization of a tectonically active Karak Thrust using radon measurement technique in the Southern Kohat Plateau, Pakistan. *J. Himal. Earth Sci.* 49 40–49.

[B35] Klindworth A. Pruesse E. Schweer T. Peplies J. Quast C. Horn M. (2013). Evaluation of general 16S ribosomal RNA gene PCR primers for classical and next-generation sequencing-based diversity studies. *Nucleic Acids Res.* 41:e1. 10.1093/nar/gks808 PMC3592464 22933715

[B36] Lane D. J. (1991). “16S/23S rRNA sequencing,” in *Nucleic Acid Techniques in Bacterial Systematics*, eds Stackebrandt E. Goodfellow M. (Chichester: Wiley), 115–175.

[B37] Laye V. J. DasSarma S. (2018). An Antarctic extreme halophile and its polyextremophilic enzyme: effects of perchlorate salts. *Astrobiology* 18 412–418. 10.1089/ast.2017.1766 29189043 PMC5910040

[B38] Leena M. C. Aamer A. S. Abdul H. Fariha H. (2018). Physiological, biochemical and phylogenetic characterization of extremely halophilic bacteria isolated from Khewra mine, Pakistan. *Appl. Ecol. Environ. Res.* 16 1243–1256. 10.15666/aeer/1602_12431256

[B39] Margesin R. Schinner F. (2001). Potential of halotolerant and halophilic microorganisms for biotechnology. *Extremophiles* 5 73–83. 10.1007/s007920100184 11354458

[B40] Maturrano L. Santos F. Rosselló-Mora R. Antón J. (2006). Microbial diversity in Maras salterns, a hypersaline environment in the Peruvian Andes. *Appl. Environ. Microbiol.* 72 3887–3895. 10.1128/AEM.02214-05 16751493 PMC1489619

[B41] Mesbah N. M. Abou-El-Ela S. H. Wiegel J. (2007). Novel and unexpected prokaryotic diversity in water and sediments of the alkaline, hypersaline lakes of the Wadi an Natrun, Egypt. *Microb. Ecol.* 54 598–617. 10.1007/s00248-006-9193-y 17450395

[B42] Mormile M. R. Biesen M. A. Gutierrez M. C. Ventosa A. Pavlovich J. B. Onstott T. C. (2003). Isolation of *Halobacterium salinarum* retrieved directly from halite brine inclusions. *Environ. Microbiol.* 5 1094–1102. 10.1046/j.1462-2920.2003.00509.x 14641589

[B43] Muyzer G. L. DeWaal E. C. Uitterlinden A. G. (1993). Profiling of complex microbial populations by denaturing gradient gel electrophoresis analysis of polymerase chain reaction-amplified genes coding for 16S rRNA. *Appl. Environ. Microbiol.* 6659 695–700. 10.1128/aem.59.3.695-700.1993 PMC202176 7683183

[B44] Nafees M. Khan N. Rukh S. Bashir A. (2013). Analysis of rock and sea salt for various essential S and inorganic. *J. Sci. Technol.* 37 9–20.

[B45] Naghoni A. Emtiazi G. Amoozegar M. A. Cretoiu M. S. Stal L. J. Etemadifar Z. (2017). Microbial diversity in the hypersaline Lake Meyghan, Iran. *Sci. Rep.* 7:11522. 10.1038/s41598-017-11585-3 28912589 PMC5599592

[B46] Narasingarao P. Podell S. Ugalde J. A. Brochier-Armanet C. Emerson J. B. Brocks J. J. (2012). De novo metagenomic assembly reveals abundant novel major lineage of Archaea in hypersaline microbial communities. *ISME J.* 6 81–93. 10.1038/ismej.2011.78 21716304 PMC3246234

[B47] Oren A. (2012). The function of gas vesicles in halophilic archaea and bacteria: theories and experimental evidence. *Life* 3 1–20. 10.3390/life3010001 25371329 PMC4187190

[B48] Oren A. (2013). Life at high salt concentrations, intracellular KCl concentrations, and acidic proteomes. *Front. Microbiol.* 4:315. 10.3389/fmicb.2013.00315 24204364 PMC3817357

[B49] Oren A. (2014). Taxonomy of halophilic Archaea: current status and future challenges. *Extremophiles* 18 825–834. 10.1007/s00792-014-0654-9 25102811

[B50] Pasteris J. D. Freeman J. J. Wopenka B. Qi K. Ma Q. Wooley K. L. (2006). What a grain of salt: what halite has to offer to discussions on the origin of life. *Astrobiology* 6 625–643. 10.1089/ast.2006.6.625 16916287

[B51] Paul D. Kumbhare S. V. Mhatre S. S. Chowdhury S. P. Shetty N. P. Marathe S. A. (2016). Exploration of microbial diversity and community structure of Lonar Lake: the only hypersaline meteorite Crater Lake within basalt rock. *Front. Microbiol.* 6:1553. 10.3389/fmicb.2015.01553 26834712 PMC4722114

[B52] Purkhold U. Pommerening-röser A. Schmid M. C. Koops H. Juretschko S. Wagner M. (2000). Phylogeny of all recognized species of ammonia oxidizers based on comparative 16S rRNA and amoA sequence analysis: implications for molecular diversity surveys. *Appl. Environ. Microbiol.* 66 5368–5382. 10.1128/AEM.66.12.5368-5382.2000.Updated 11097916 PMC92470

[B53] Ram M. N. Oren A. Papke R. T. (2016). Analysis of the bacteriorhodopsin-producing haloarchaea reveals a core community that is stable over time in the salt crystallizers of Eilat, Israel. *Extremophiles* 20 747–757. 10.1007/s00792-016-0864-4 27444744

[B54] Reiser R. Tasch P. (1960). Investigation of the viability of osmophile bacteria of great age. *Trans. Kans. Acad. Sci.* 63 31–34. 13740659

[B55] Robinson C. K. Wierzchos J. Black C. Crits-Christoph A. Ma B. Ravel J. (2015). Microbial diversity and the presence of algae in halite endolithic communities are correlated to atmospheric moisture in the hyper-arid zone of the Atacama Desert. *Environ. Microbiol.* 17 299–315. 10.1111/1462-2920.12364 24372972

[B56] Roohi A. Ahmed I. Khalid N. Iqbal M. Jamil M. (2014). Isolation and phylogenetic identification of halotolerant/halophilic bacteria from the salt mines of Karak, Pakistan. *Int. J. Agric. Biol.* 16 564–570.

[B57] Ruvindy R. White R. A. Neilan B. A. Burns B. (2016). Unravelling core microbial metabolisms in the hypersaline microbial mats of Shark Bay using high-throughput metagenomics. *ISME J.* 10 183–196. 10.1038/ismej.2015.87 26023869 PMC4681862

[B58] Shah M. Hasan F. Hashmi S. S. Muhammad W. Zubair M. Asad Ullah M. (2018). Diversity of halophiles in Karak salt mine, KP, Pakistan and their ability to produce enzyme of industrial importance. *Int. J. Biosci.* 13 257–266. 10.12692/ijb/13.1.257-266

[B59] Shah W. Nawab A. Shahbaz A. Muhammad Q. Hazir R. Sami U. (2017). Molecular characterization and growth optimization of halo-tolerant amylase producing bacteria isolated from salt mines of Karak. *Pure Appl. Biol.* 6 385–393. 10.19045/bspab.2017.60038

[B60] Sharif Q. M. Hussain M. Hussain T. M. (2007). Chemical evaluation of major salt deposits of Pakistan. *J. Chem. Soc. Pak.* 29 569–574.

[B61] Singer E. Webb E. A. Nelson W. C. Heidelberg J. F. Ivanova N. Pati A. (2011). Genomic potential of *Marinobacter aquaeolei*, a biogeochemical ‘Opportunitroph’. *Appl. Environ. Microbiol.* 77 2763–2771. 10.1128/AEM.01866-10 21335390 PMC3126349

[B62] Stepanov V. G. Xiao Y. Lopez A. J. Roberts D. J. Fox G. E. (2016). Draft genome sequence of *Marinobacter* sp. strain P4B1, an electrogenic perchlorate-reducing strain isolated from a long-term mixed enrichment culture of marine bacteria. *Genome Announc.* 4:01617-15. 10.1128/genomeA.01617-15 26798109 PMC4722276

[B63] Vargas V. A. Delgado O. D. Hatti-Kaul R. Mattiasson B. (2004). Lipase- producing microorganisms from a Kenyan alkaline soda lake. *Biotechnol. Lett.* 26 81–86. 10.1023/B:BILE.0000012898.50608.12 15000472

[B64] Vavourakis C. D. Ghai R. Rodriguez-Valera F. Sorokin D. Y. Tringe S. G. Hugenholtz P. (2016). Metagenomic insights into the uncultured diversity and physiology of microbes in four hypersaline soda lake brines. *Front. Microbiol.* 7:211. 10.3389/fmicb.2016.00211 26941731 PMC4766312

[B65] Ventosa A. Fernández A. B. León M. J. Sánchez-Porro C. Rodriguez-Valera F. (2014). The Santa Pola saltern as a model for studying the microbiota of hypersaline environments. *Extremophiles* 18 811–824. 10.1007/s00792-014-0681-6 25129545

[B66] Vreeland R. H. Piselli A. F. McDonnough S. Meyers S. S. (1998). Distribution and diversity of halophilic bacteria in a subsurface salt formation. *Extremophiles* 2 321–331. 10.1007/s007920050075 9783180

[B67] Welsh D. T. (2000). Ecological significance of compatible solute accumulation by micro-organisms: from single cells to global climate. *FEMS Microbiol.* 24 263–290. 10.1111/j.1574-6976.2000.tb00542.x 10841973

[B68] Xiao W. Wang Z. Wang Y. (2013). Comparative molecular analysis of the prokaryotic diversity of two salt mine soils in southwest China. *J. Basic Microbiol.* 5 942–952. 10.1002/jobm.201200200 23457089

[B69] Yadav A. N. Sharma D. Gulati S. Singh S. Dey R. Pal K. K. (2015). Haloarchaea endowed with phosphorus solubilization attribute implicated in phosphorus cycle. *Sci. Rep.* 5:12293. 10.1038/srep12293 26216440 PMC4516986

[B70] Yau S. Lauro F. M. Williams T. J. Demaere M. Z. Brown M. V. Rich J. (2013). Metagenomic insights into strategies of carbon conservation and unusual sulfur biogeochemistry in a hypersaline Antarctic lake. *ISME J.* 7 1944–1961. 10.1038/ismej.2013.69 23619305 PMC3965305

[B71] Yildiz E. Ozcan B. Caliskan M. (2012). Isolation, characterization and phylogenetic analysis of halophilic archaea from a salt mine in central Anatolia (Turkey). *Pol. J. Microbiol.* 61 111–117. 10.33073/pjm-2012-014 23163210

