# Supplementary figures and images for: Metagenomic Insights Into the Diversity of Halophilic Microorganisms Indigenous to the Karak Salt Mine, Pakistan
Source: Front Microbiol. 2020 Jul 14;11:1567. doi: 10.3389/fmicb.2020.01567 (PMC7386132; doi:10.3389/fmicb.2020.01567)

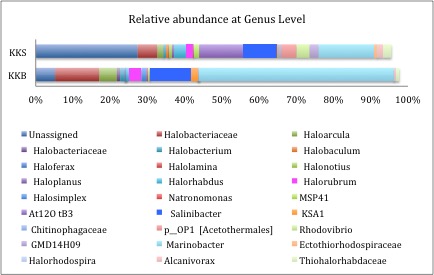

Supplement: FIGURE S1 — Relative distribution of halophilic communities in brine and salt samples of Karak salt mine plotted at low-level tax. Sequences that were not allocated to any phylum are depicted as “unassigned.” [file Image_1.JPEG]
